# Effects of different mesenchymal stromal cell sources and delivery routes in experimental emphysema

**DOI:** 10.1186/s12931-014-0118-x

**Published:** 2014-10-03

**Authors:** Mariana A Antunes, Soraia C Abreu, Fernanda F Cruz, Ana Clara Teixeira, Miquéias Lopes-Pacheco, Elga Bandeira, Priscilla C Olsen, Bruno L Diaz, Christina M Takyia, Isalira PRG Freitas, Nazareth N Rocha, Vera L Capelozzi, Débora G Xisto, Daniel J Weiss, Marcelo M Morales, Patricia RM Rocco

**Affiliations:** Laboratory of Pulmonary Investigation, Carlos Chagas Filho Biophysics Institute, Centro de Ciências da Saúde, Federal University of Rio de Janeiro, Avenida Carlos Chagas Filho, s/n, Bloco G-014, Ilha do Fundão – 21941-902, Rio de Janeiro, RJ Brazil; Laboratory of Cellular and Molecular Physiology, Federal University of Rio de Janeiro, Rio de Janeiro, Brazil; Laboratory of Inflammation, Federal University of Rio de Janeiro, Rio de Janeiro, Brazil; Laboratory of Cellular Pathology, Federal University of Rio de Janeiro, Rio de Janeiro, Brazil; Laboratory of Cellular and Molecular Cardiology, Federal University of Rio de Janeiro, Rio de Janeiro, Brazil; Fluminense Federal University, Niteroi, Rio de Janeiro Brazil; Department of Pathology, University of São Paulo, São Paulo, Brazil; Department of Medicine, University of Vermont, Vermont, USA

**Keywords:** Elastase, Emphysema, Remodeling, Macrophage, Mesenchymal stromal cells

## Abstract

We sought to assess whether the effects of mesenchymal stromal cells (MSC) on lung inflammation and remodeling in experimental emphysema would differ according to MSC source and administration route. Emphysema was induced in C57BL/6 mice by intratracheal (IT) administration of porcine pancreatic elastase (0.1 UI) weekly for 1 month. After the last elastase instillation, saline or MSCs (1×10^5^), isolated from either mouse bone marrow (BM), adipose tissue (AD) or lung tissue (L), were administered intravenously (IV) or IT. After 1 week, mice were euthanized. Regardless of administration route, MSCs from each source yielded: 1) decreased mean linear intercept, neutrophil infiltration, and cell apoptosis; 2) increased elastic fiber content; 3) reduced alveolar epithelial and endothelial cell damage; and 4) decreased keratinocyte-derived chemokine (KC, a mouse analog of interleukin-8) and transforming growth factor-β levels in lung tissue. In contrast with IV, IT MSC administration further reduced alveolar hyperinflation (BM-MSC) and collagen fiber content (BM-MSC and L-MSC). Intravenous administration of BM- and AD-MSCs reduced the number of M1 macrophages and pulmonary hypertension on echocardiography, while increasing vascular endothelial growth factor. Only BM-MSCs (IV > IT) increased the number of M2 macrophages. In conclusion, different MSC sources and administration routes variably reduced elastase-induced lung damage, but IV administration of BM-MSCs resulted in better cardiovascular function and change of the macrophage phenotype from M1 to M2.

## Introduction

Emphysema, a key feature of chronic obstructive pulmonary disease (COPD), is characterized by the enlargement of air spaces accompanied by destruction of parenchymal structure and impaired pulmonary regeneration [1]. Currently, COPD is the fourth leading cause of death worldwide, and so far there has been no effective therapy for patients with emphysema [2]. One potential therapeutic approach for emphysema has focused on inducing lung repair and regeneration and/or decreasing chronic inflammation by administering mesenchymal stem (stromal) cells (MSCs) of bone marrow or adipose origin [3]. A number of preclinical studies have shown that MSCs attenuate lung inflammation and apoptosis in experimental emphysema [4-7]. Furthermore, a recent clinical study showed that the intravenous (IV) administration of non HLA-matched allogeneic bone marrow MSCs in emphysema patients is safe; however, no functional improvement was reported, although a decrease in an inflammatory mediator, C-reactive protein, was observed in treated patients [8]. Nevertheless, depending on the site of origin, MSCs may have different phenotypes, including differences in immunogenicity, anti-inflammatory and regenerative activity, and expansibility in culture [9,10], which may lead to differing results depending on MSC source. Therefore, further comparative experimental studies are required to better assess the efficacy of different sources of MSCs for use in emphysema.

A further critical aspect for cell transplantation is the selection of the optimal administration route. While IV injection of MSCs is generally utilized in preclinical studies of experimental emphysema, due to ease of administration and subsequent wide biodistribution [4,6,11,12], intratracheal (IT) administration of MSCs also attenuates lung damage [13,14]. Thus, no definite conclusion has been reached regarding the optimal administration route of MSCs in experimental emphysema.

The aims of the present study were to: (a) comparatively assess the extent to which different sources (bone marrow, adipose, or lung tissue) of MSCs are able to decrease inflammation and promote alveolar epithelium and endothelium repair, thereby improving lung function in elastase-induced emphysema in mice, (b) investigate whether IV versus IT administration of MSCs influences their effectiveness on lung inflammation and remodeling, and (c) evaluate the effects of IV versus IT delivery of the aforementioned different sources of MSCs on elastase (emphysema)-induced changes in cardiac function.

## Materials and methods

This study was approved by the Ethics Committee of the Health Sciences Centre, Federal University of Rio de Janeiro. All animals received humane care in compliance with the “Principles of Laboratory Animal Care” formulated by the National Society for Medical Research and the U.S. National Research Council “Guide for the Care and Use of Laboratory Animals”.

### Isolation and culture of bone marrow-, adipose tissue-, and lung tissue-derived MSCs

Ten male C57BL/6 mice (weight 20–25 g, age 2 months) were used as donors. Bone marrow cells were obtained from femurs and tibias. After isolation, 1 × 10^7^ bone marrow-derived cells were cultured (37°C, 5% CO_2_) in T25 culture flasks (TPP, Schaffhausen, Switzerland) with Dulbecco’s Modified Eagle Medium (DMEM; Invitrogen, CA, USA) containing 15 mM HEPES (Sigma, MO, USA), 15% inactivated fetal bovine serum (FBS) (Invitrogen, CA, USA), 100 units/mL penicillin, and 100 mg/mL streptomycin antibiotic solution (Gibco, NM, USA). MSCs from lung and adipose tissue (epididymal fat pad) were obtained as previously described [15]. Tissues were collected, rinsed in PBS, transferred to a Petri dish, and cut into small pieces. The dissected pieces (around 0.2-0.8 cm^3^) were washed with PBS, cut into smaller fragments, and subsequently digested with collagenase type I (1 mg/mL in DMEM/10 mM HEPES) for 30 minutes to 1 hour at 37°C. Whenever gross remnants persisted after collagenase digestion were allowed to settle for 1 to 3 minutes, and the supernatant was transferred to a new tube containing fresh medium and centrifuged at 400 g for 10 minutes at room temperature (RT). The pellets were re-suspended in 3.5 mL D-MEM containing 1% antibiotic-antimycotic solution DMEM (Invitrogen, CA, USA), seeded in six-well dishes (3.5 mL/well), and incubated at 37°C in a humidified atmosphere containing 5% CO_2_. On day 3 of culture, the medium was changed and non-adherent cells were removed. Adherent cells reaching 80% confluence were passaged with 0.05% trypsin-EDTA solution (Gibco, NM, USA) and then maintained in DMEM with 10% FBS (complete medium).

At the third passage, approximately 1 × 10^6^ cells were characterized as MSCs according to the International Society of Cellular Therapy Consensus, i.e., adherent to plastic under standard conditions, expressing some surface markers (CD73, CD90 and CD105) and lacking expression of others (CD34, CD45, CD11b, CD19), and demonstrating capacity to differentiate into mesenchymal lineages under *in vitro* conditions [16]. Flow cytometry used antibodies against CD45 (leukocytes), CD34 (hematopoietic precursors), CD29 and CD45 (non-hematopoietic precursors), and Sca-1 (stem/progenitor cells) (BD Biosciences, USA). The absence of CD34 and CD45 and the presence of CD29, and Sca-1 were used to identify MSCs [17]. To measure the small-angle forward scatter (FSC) intensity (~0°–5°) and the limited-angle side scatter (SSC) intensity (~85°–95°), a photodiode and a photomultiplier tube were used respectively. The different MSC populations were further characterized by their capacity to differentiate into osteoblasts and chondroblasts. Osteogenic differentiation was induced by culturing MSCs for up to 3 weeks in D-MEM 10% FBS and 15 mM HEPES (Sigma, MO, USA), supplemented with 10–8 M/l dexamethasone (Sigma, MO, USA), 5 μg/mL ascorbic acid 2-phosphate (Sigma, MO, USA), and 10 mM/l β-glycerolphosphate (Sigma MO, USA). To observe calcium deposition, cultures were stained with Alizarin Red S (Nuclear, SP, Brazil). To induce chondrogenic differentiation, MSCs were cultured in DMEM supplemented with 10 ng/mL TGF-β1 (Sigma, MO, USA), 50 nM ascorbic acid 2-phosphate (Sigma, MO, USA), and 6.25 mg/mL insulin for 3 weeks. To confirm differentiation, cells were fixed with 4% paraformaldehyde in PBS for 1 hour at RT and stained with Alcian Blue pH 2.5.

### Animal preparation and experimental protocol

**Figure 1:**
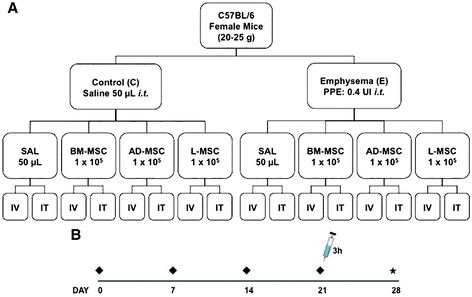
**Schematic flow chart (** **A** **) and timeline (** **B** **) of the study design.** C: intratracheal instillation of 50 μL of saline, E: intratracheal instillation of 0.1 UI of pancreatic porcine elastase (PPE), SAL: injection of 50 μL of saline, BM-MSC: bone marrow mesenchymal stromal cell (1 × 10^5^) administration; AD-MSC: adipose tissue derived mesenchymal stromal cell (1 × 10^5^) administration; L-MSC: lung derived mesenchymal stromal cell (1 × 10^5^) administration; IV/IT: intravenous or intratracheal injection 3 h after the last instillation of saline or PPE; ♦: saline or PPE instillation; ★: all data were analyzed at day 28.

C57BL/6 mice (weight: 20–25 g, age 2 months) were randomly assigned to two main groups: control (C) and emphysema (E). In group E, mice received IT pancreatic porcine elastase (0.1 UI PPE in 50 μL saline) once a week for 4 weeks [11], while group C received saline (50 μL) using the same protocol. Three hours after the last instillation, animals in the C and E groups were further randomized to receive saline solution (0.9% NaCl, 50 μL, SAL), bone marrow MSCs (BM-MSC, 1 × 10^5^ in 50 μL saline), adipose MSCs (AD-MSC, 1 × 10^5^ in 50 μL saline), or lung MSCs (L-MSC, 1 × 10^5^ in 50 μL saline) by the IV or IT route (Figure 1). As we have previously observed that administration of a control cell population (lung fibroblasts) had no effect on the experimental endpoints in this model, this arm was not included in the present study. Briefly, mice were anesthetized with sevoflurane and either the left jugular vein (for IV administration) or the trachea (for IT administration) of each mouse was exposed by ventral neck dissection and MSCs slowly injected over a period of 2 min.

### Echocardiography

For echocardiographic assessment of cardiac function, three mice per experimental group were anesthetized with isoflurane 1.5%, shaved over the precordial region, and examined with a Vevo 770 apparatus (VisualSonics, Toronto, ON, Canada) coupled to a 30 MHz transducer. Images were obtained from the parasternal view. M-mode images showed left ventricular muscle thickness. One long-axis and four short-axis B-dimensional views of both ventricles were acquired to calculate the left and right ventricular areas [18]. Pulsed-wave Doppler was used to measure pulmonary artery acceleration time (PAT), and pulmonary artery ejection time (PET) [19,20]. All parameters followed American Society of Echocardiography and European Association of Cardiovascular Imaging recommendations.

### Mechanical parameters

One week after therapy, the animals were sedated (diazepam 1 mg i.p.), anesthetized (thiopental sodium 20 mg/kg i.p.), tracheotomized, paralyzed (vecuronium bromide, 0.005 mg/kg i.v.), and ventilated with a constant flow ventilator (Samay VR15; Universidad de la Republica, Montevideo, Uruguay) set to the following parameters: rate 100 breaths/min, tidal volume (V_T_) 0.2 mL, and fraction of inspired oxygen (FiO_2_) 0.21. The anterior chest wall was surgically removed and a positive end-expiratory pressure of 2 cmH_2_O applied. Airflow and tracheal pressure (Ptr) were measured. Lung mechanics were analyzed by the end-inflation occlusion method. In an open chest preparation, Ptr reflects transpulmonary pressure (P_L_). Static lung elastance (Est, L) was determined by dividing elastic recoil pressure (Pel) by V_T_. Lung mechanics parameters were measured 10 times in each animal. All data were analyzed using ANADAT software (RHT-InfoData, Inc., Montreal, Quebec, Canada). All experiments lasted less than 15 min.

### Lung histology

At the end of the experiment, laparotomy was performed and heparin (1000 IU) injected into the vena cava. The trachea was clamped at end-expiration, and the abdominal aorta and vena cava were sectioned, producing massive hemorrhage and terminal bleeding for euthanasia. The right lung was then removed, fixed in 3% buffered formalin, and embedded in paraffin; 4-μm-thick slices were cut and stained with hematoxylin–eosin. Lung histology analysis was performed with an integrating eyepiece with a coherent system consisting of a grid with 100 points and 50 lines of known length coupled to a conventional light microscope (Olympus BX51, Olympus Latin America-Inc., Brazil). The volume fraction of hyperinflated, collapsed, and normal pulmonary areas, the mean linear intercept (Lm), and the percentage of neutrophils in pulmonary tissue were determined by the point-counting technique across 10–20 random, non-coincident microscopic fields [21,22]. Collagen (Picrosirius polarization method) and elastic fibers (Weigert’s resorcin fuchsin method with oxidation) were computed around the small airways and in the lung parenchyma, respectively, using Image-Pro Plus 6.0 software [23].

### Immunohistochemistry

Immunohistochemistry for macrophage subpopulations (M1 and M2 phenotypes) in lung tissue was done using iNOS rabbit anti-mouse polyclonal antibody (M1, catalog no. Rb-9242, Thermo Scientific) and arginase-1 rabbit anti-mouse polyclonal antibody (M2, catalog no. sc-20150, Santa Cruz Biotechnology). Antibodies were detected using a secondary antibody labeled with peroxidase (Histofine mouse MAX PO anti-rat and anti-rabbit, Nichirei Biosciences, Tokyo, Japan) followed by the chromogen substrate diaminobenzidine (Liquid DAB, Dakocytomation, USA, catalog no. K3468). Analysis was performed in 30 images of high-power fields (×400 magnification) per slide, taken with an Evolution VR Cooled Color 13-bit digital camera (Media Cybernetics, Canada) and manually selected under a light microscope (Nikon Eclipse 400, Nikon Instruments Tokyo, Japan). The areas occupied by nucleated macrophages and cells with positive staining for the phenotype marker in each tissue area were then calculated and expressed as fractional area occupied by positive cells. The images were analyzed using Image Pro Plus 4.5.1 software (Media Cybernetics).

### Enzyme-linked immunosorbent assay (ELISA)

Levels of keratinocyte-derived chemokine (KC, a mouse analog of interleukin-8), transforming growth factor (TGF)-β, and vascular endothelial growth factor (VEGF) in lung tissue were evaluated by ELISA using matched antibody pairs from PrepoTech and R & D Systems (Minneapolis, MN, USA), according to manufacturer instructions. Results are expressed as pg/mL.

### Transmission electron microscopy (TEM)

Three slices (2 × 2 × 2 mm) were cut from three different segments of the left lung and fixed in glutaraldehyde 2.5% and phosphate buffer 0.1 M (pH = 7.4) for electron microscopy (JEOL 1010 Transmission Electron Microscope, Tokyo, Japan). On each lung electron microscopy image (20 fields/animal), the following alterations were analyzed: alveolar-capillary membrane damage, type II pneumocyte lesion, and endothelial cell lesion [24]. Pathologic findings were graded on a five-point, semiquantitative, severity-based scoring system as follows: 0 = normal lung parenchyma, 1 = changes in 1–25%, 2 = changes in 26–50%, 3 = changes in 51–75%, and 4 = changes in 76–100% of examined tissue.

### Apoptosis assay of lung

Terminal deoxynucleotidyl transferase biotin-dUTP nick end labeling (TUNEL) staining was used to assay cellular apoptosis [25]. Ten fields per section from regions with cell apoptosis were examined at × 400 magnification. A five-point, semiquantitative, severity-based scoring system was used to assess the degree of apoptosis: 0 = normal lung parenchyma; 1 = 1–25%; 2 = 26–50%; 3 = 51–75%; and 4 = 76–100% of examined tissue [24]. The pathologist or technician working on the light microscopy and TEM images was blinded to group assignment.

### Statistical analyses

One-way ANOVA followed by Tukey’s test was used to compare the different parameters for each administration route. For non-parametric results, the Kruskal-Wallis test followed by Dunn’s test was used. All tests were performed using the Prism 5.0 software package (GraphPad Software Inc., La Jolla, CA, USA), and statistical significance was established as p < 0.05.

## Results

Intravenous administration of lung-derived MSCs led to immediate death of all mice, which may be associated with the larger size of the L-MSCs (see below) or with cellular clumping resulting in pulmonary embolism. Thus, this group was not included in further analysis. Conversely, survival rate in all other groups was 100%. As no significant differences in any endpoint measures were observed between any of the C groups (Table 1), henceforth a single C group, which consists of the average of all C groups, was reported.

**Table 1:** **Characteristics of the control groups**

			**Normal (%)**	**Collapse (%)**	**Hyperinflation (%)**	**Lm (μm)**
C	SAL	IV	93.23 ± 0.90	6.77 ± 0.90	0.00 ± 0.00	33.86 ± 2.93
		IT	94.05 ± 1.98	5.95 ± 1.98	0.00 ± 0.00	33.80 ± 2.24
	BM-MSC	IV	92.10 ± 2.46	7.90 ± 2.46	0.00 ± 0.00	36.43 ± 2.53
	AD-MSC		94.53 ± 1.07	5.47 ± 1.07	0.00 ± 0.00	36.03 ± 2.28
	BM-MSC	IT	91.58 ± 3.11	8.42 ± 3.11	0.00 ± 0.00	36.44 ± 0.32
	AD-MSC		92.55 ± 1.01	7.45 ± 1.01	0.00 ± 0.00	35.96 ± 0.90
	L-MSC		92.09 ± 2.97	7.91 ± 2.97	0.00 ± 0.00	36.77 ± 1.00

Values are means (±SD) of 7 animals in each group. All values were computed in ten random, non-coincident fields per mice. Fraction area of normal, collapsed, and hyperinflated alveoli. Lm: mean linear intercept. In the control (C) group, saline was instilled intratracheally. At day 21, all groups were randomized to receive saline and bone marrow (BM), adipose (AD), or lung-derived (LD) mesenchymal stem cells (MSC, 1×10^5^ cells) intravenously (IV) or intratracheally (IT).

### MSC characterization

All MSC sources were characterized as CD19^−^/CD34^−^/CD45^−^/CD29^+^/Sca1^+^ by flow cytometry (Table 2). LD-MSCs were 10% and 24% larger in size compared to AD-MSCs and BM-MSCs, respectively. All MSC lineages were similarly capable of *in vitro* differentiation into osteoblasts and chondroblasts.

**Table 2:** **Cell characterization by flow cytometry**

	**BM-MSC**	**AD-MSC**	**L-MSC**
CD19^−^	99.29%	99.74%	99.98%
CD29^+^	99.00%	99.48%	99.63%
CD34^−^	96.76%	98.30%	99.62%
CD45^−^	95.38%	98.40%	88.77%
Sca1^+^	58.46%	53.29%	38.76%

BM-MSC: bone marrow-derived mesenchymal stem (stromal) cells; AD-MSC: adipose tissue-derived mesenchymal stem (stromal) cells; L-MSC: lung tissue-derived mesenchymal stem (stromal) cells. Flow cytometry reveals that mesenchymal stem cells are negative (^−^) for leukocyte (CD45), hematopoietic (CD34), and B-cells (CD19), while they are concomitantly positive (^+^) for stem cell (Sca1) and mesenchymal stem cell markers (CD29).

### Development of emphysema model induced by repeated elastase doses

**Figure 2:**
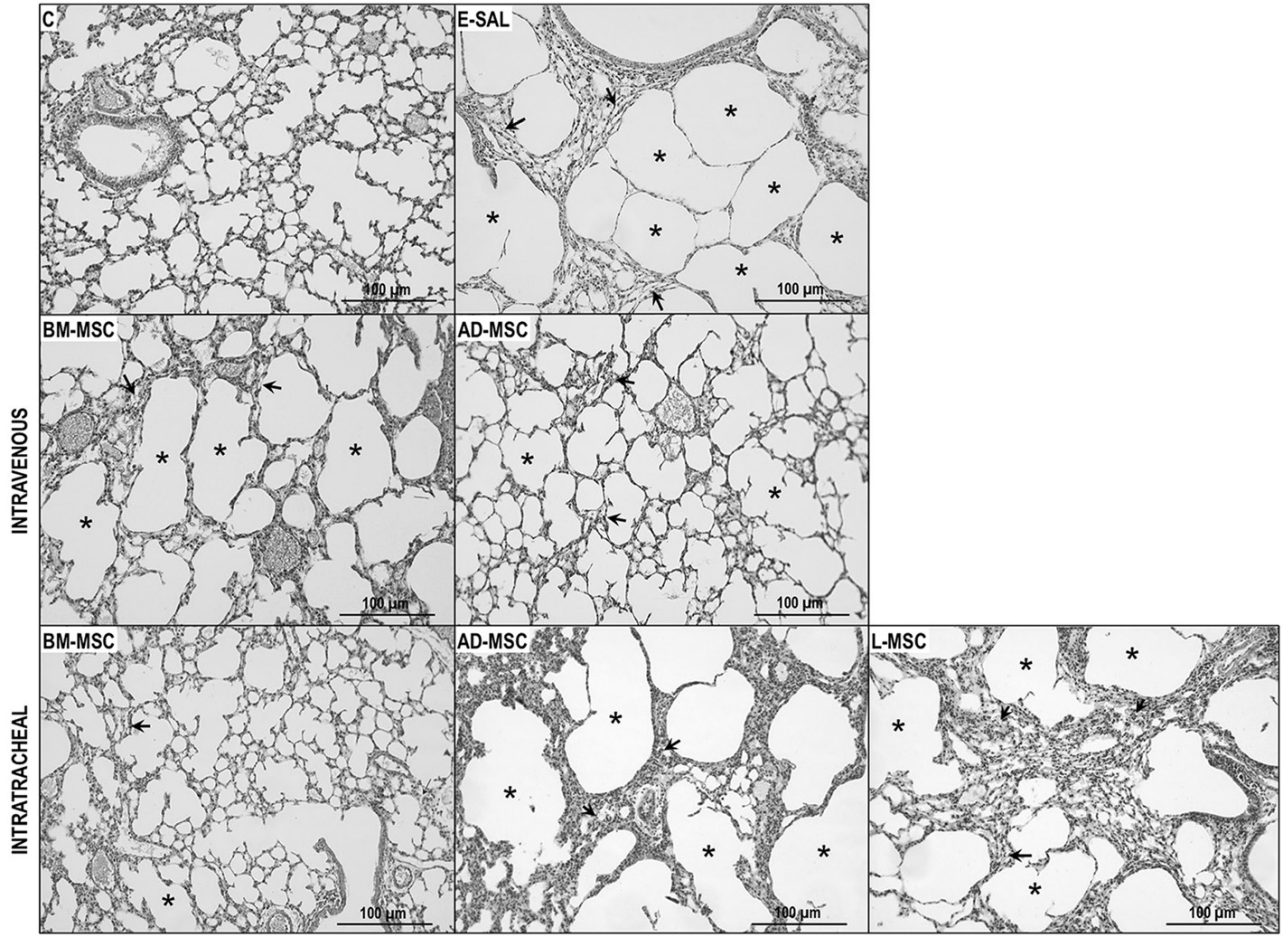
**Representative photomicrographs of the lung parenchyma.** C: control groups. E: emphysema groups. Mice were treated with saline (SAL) or bone marrow (BM), adipose (AD), and lung (L)-derived mesenchymal stromal cells (MSC). IV: intravenous route. IT: intratracheal route.

**Figure 3:**
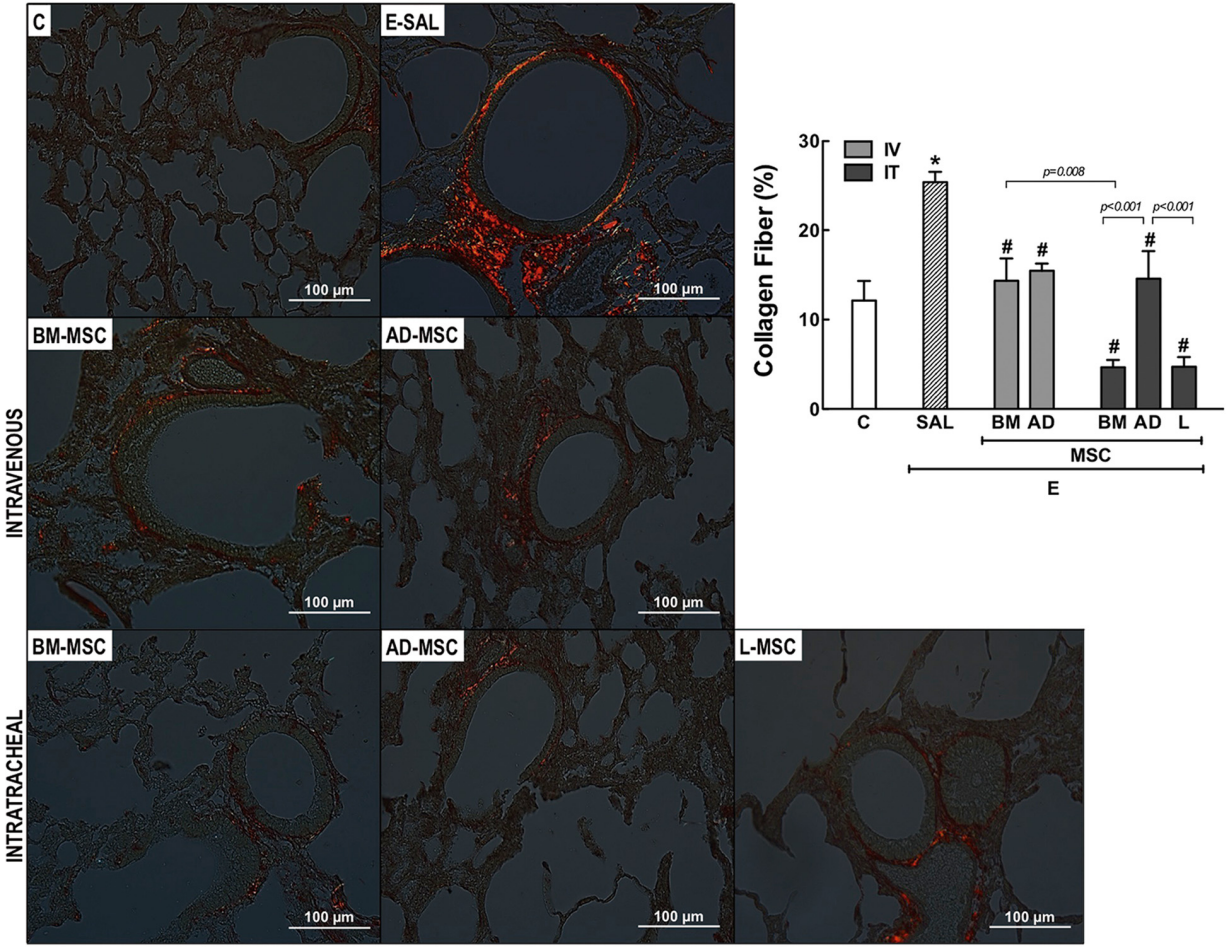
**Collagen fibers in small airways (Picrosirius-polarization method).** C: control groups. E: emphysema groups. Mice were treated with saline (SAL) or bone marrow (BM), adipose (AD), and lung (L)-derived mesenchymal stromal cells (MSC). IV: intravenous route. IT: intratracheal route. Values are mean ± SD of 7–30 mice in each group. All values were computed in ten random, non-coincident fields per animal. *Vs. C group (p < 0.05). ^#^Vs. E-SAL group (p < 0.05).

**Figure 4:**
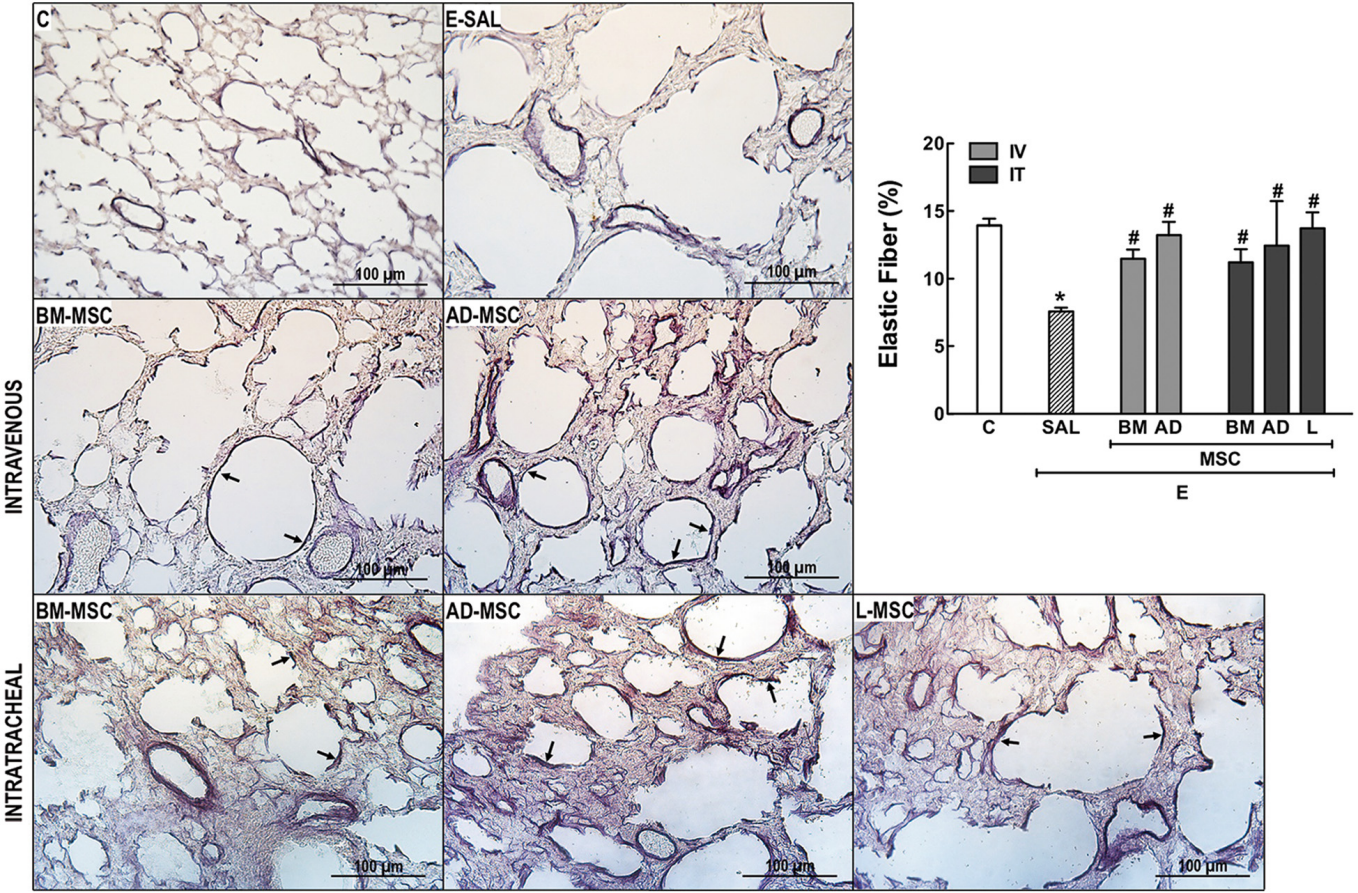
**Elastic fibers in the alveolar septa (Weigert’s resorcin fuchsin method).** C: control groups. E: emphysema groups. Mice were treated with saline (SAL) or bone marrow (BM), adipose (AD), and lung (L)-derived mesenchymal stromal cells (MSC). IV: intravenous route. IT: intratracheal route. Values are mean ± SD of 7–30 mice in each group. All values were computed in ten random, non-coincident fields per animal. *Vs. C group (p < 0.05). ^#^Vs. E-SAL group (p < 0.05).

**Figure 5:**
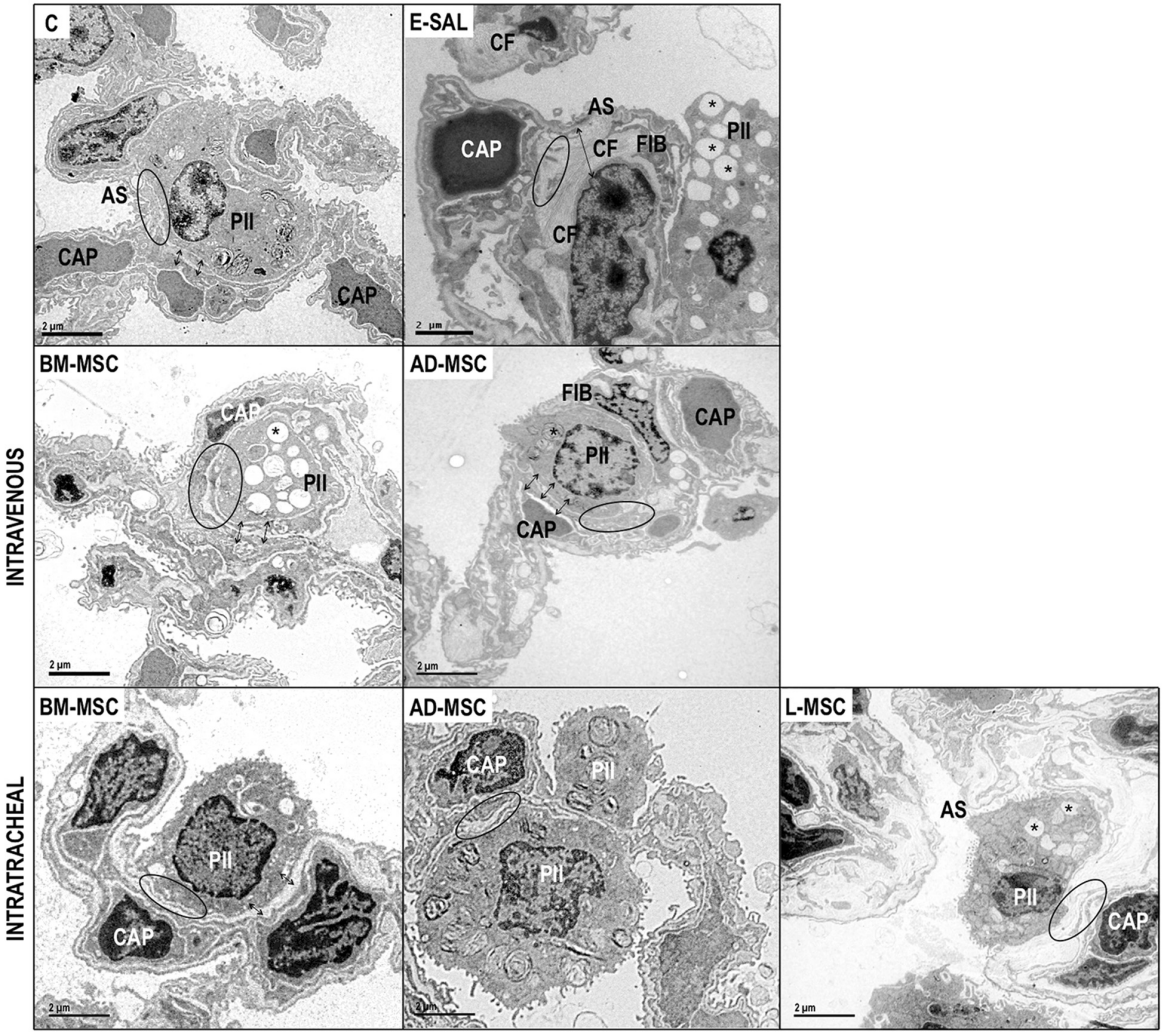
**Electron microscopy of lung parenchyma.** C: control groups. E: emphysema groups. Mice were treated with saline (SAL), or bone marrow (BM), adipose (AD) and lung (L)-derived mesenchymal stem cells (MSC). In the C group, the alveolar epithelium is formed by type II pneumocytes (PII). Alveolar septa (AS) and capillaries (CAP) are intact. In E-SAL group, the AS is ruptured with capillary loss (arrows) and shows fibroblasts (FIB) and increase in collagen fibers. The alveolar epithelium was apparently normal but had zones with AS thickness containing no capillaries (arrows) even after intravenous or intratracheal BM-MSC and AD-MSC administration. After intratracheal L-MSC treatment, the AS is restored with new capillaries and collagen fibers are diminished.

**Figure 6:**
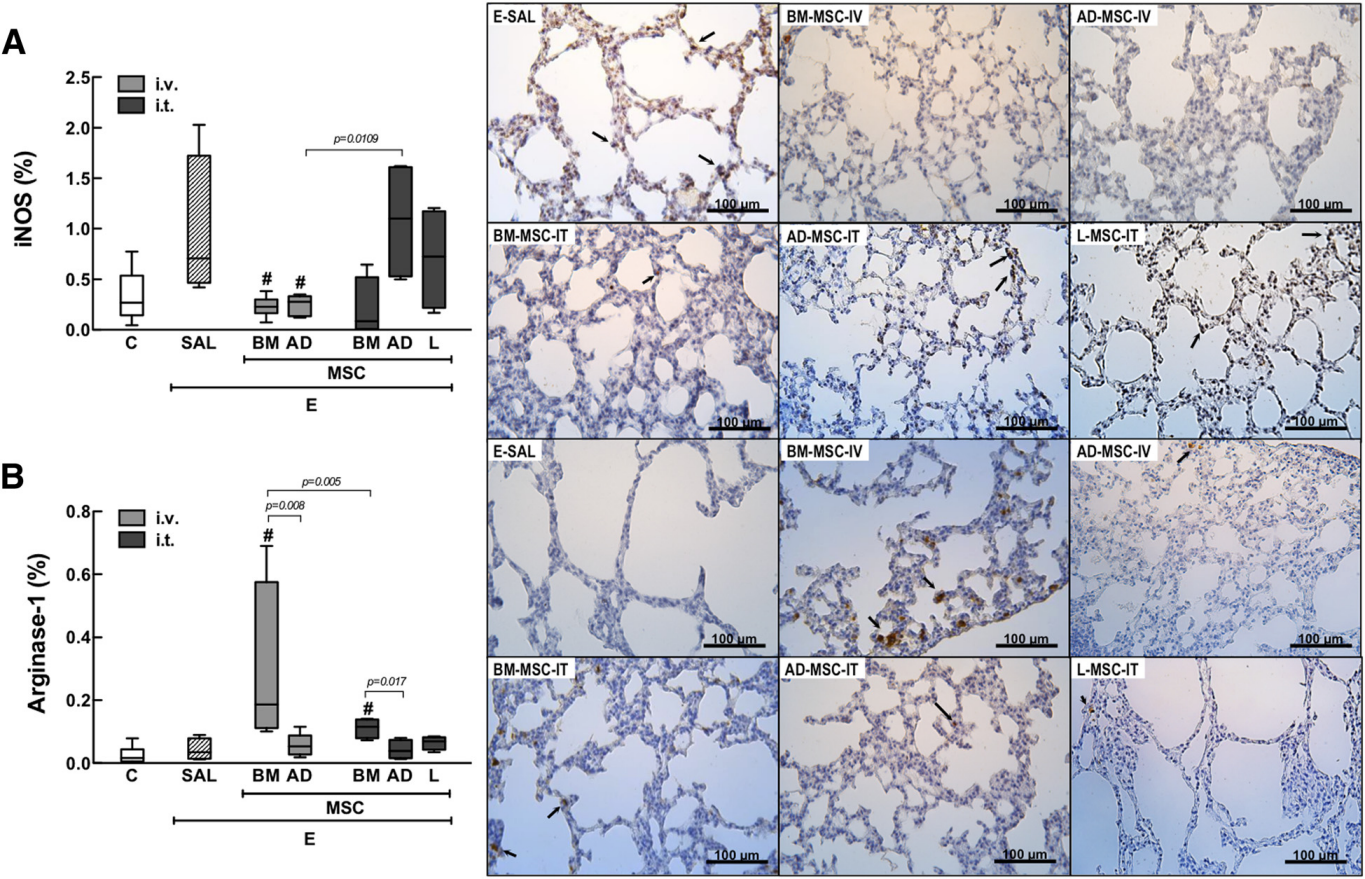
**Immunohistochemistry for iNOS (** **A** **) and arginase-1 (** **B** **).** C: control groups. E: emphysema groups. Mice were treated with saline (SAL), or bone marrow (BM), adipose (AD) and lung (L)-derived mesenchymal stem cells (MSC). IV: intravenous route. IT: intratracheal route. Values are mean ± SD of 5–30 mice in each group. All values were computed in ten random, non-coincident fields per animal. *Vs. C group (p < 0.05). ^#^Vs. E-SAL group (p < 0.05). Note positive cells in brown (arrow).

**Figure 7:**
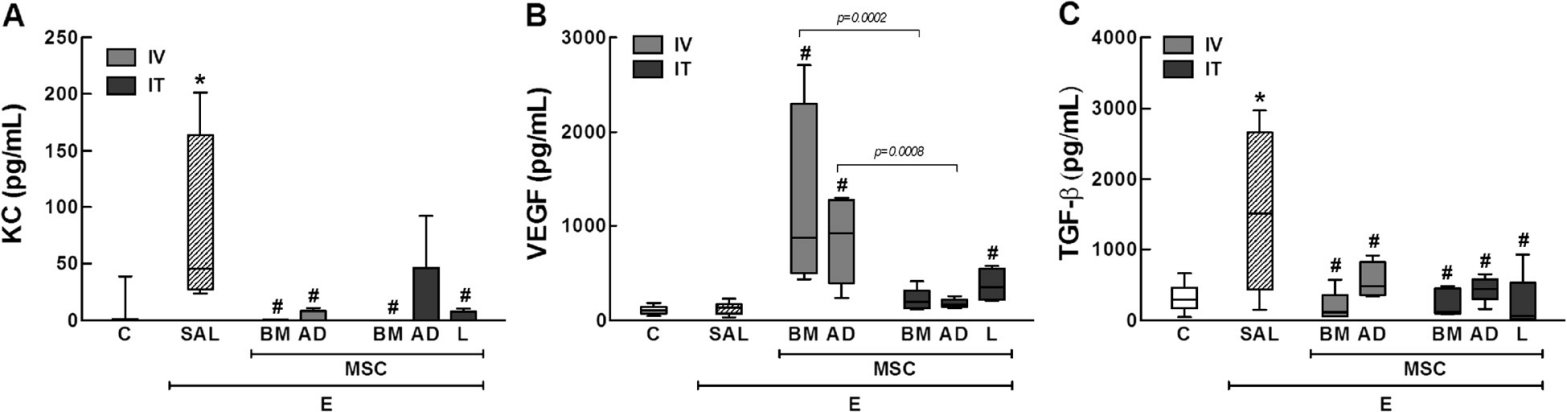
**Levels of KC (** **A** **), VEGF (** **B** **), and TGF-β (** **C** **) in lung tissue.** C: control groups. E: emphysema groups. Mice were treated with saline (SAL), or bone marrow (BM), adipose (AD) and lung (L)-derived mesenchymal stem cells (MSC). IV: intravenous route. IT: intratracheal route. Values are mean ± SD of 5–30 mice in each group. All values were computed in ten random, non-coincident fields per animal. *Vs. C group (p < 0.05). ^#^Vs. E-SAL group (p < 0.05).

In the E-SAL group, the fractional area of alveolar collapse, hyperinflation, and neutrophils in lung tissue (Table 3, Figure 2), collagen fiber content around the small airways (Figure 3), and lung cell apoptosis (Table 4) were increased compared to C group, whereas the amount of elastic fibers was reduced (Figure 4). Ultrastructural analysis of lung parenchyma in E-SAL animals demonstrated the presence of alveolar-capillary membrane lesions, as well as type II epithelial and endothelial cell damage (Table 4, Figure 5). In E-SAL animals, the number of parenchymal macrophages with the M1 immunophenotype was increased (Figure 6) with no significant changes in numbers of macrophages with the M2 immunophenotype. KC, VEGF, and TGF-β levels in lung tissue were higher in E-SAL than in C animals (Figure 7). No significant changes in Est, L were observed between the E-SAL and C groups (Table 5).

**Table 3:** **Lung morphometry and cellularity**

			**Normal (%)**	**Collapse (%)**	**Hyperinflation (%)**	**Lm (μm)**	**Neutrophils (%)**
C			91.25 ± 3.91	8.35 ± 3.61	0.40 ± 1.18	35.92 ± 2.55	1.45 ± 0.93
E	SAL		45.34 ± 10.02*	27.73 ± 13.55*	26.93 ± 12.32*	60.08 ± 6.37*	6.28 ± 0.76*
	BM-MSC	IV	55.09 ± 11.99*	15.19 ± 3.17*^#^	29.73 ± 12.20*	41.45 ± 5.37^**#**^	1.80 ± 0.70^**#**^
	AD-MSC		69.33 ± 10.74*^#^	9.70 ± 4.48^**#**^	21.33 ± 9.01*	38.85 ± 4.25^**#**^	1.26 ± 0.76^**#**^
	BM-MSC	IT	69.03 ± 5.18*^#^	17.21 ± 4.37*	13.77 ± 5.29*^#†^	38.39 ± 1.20^**#**^	1.39 ± 0.10^**#**^
	AD-MSC		64.01 ± 14.43*^#^	13.32 ± 4.12^**#**^	22.67 ± 13.10*	35.10 ± 0.82^**#**^	0.91 ± 0.12^**#**^
	L-MSC		66.91 ± 8.53*^#^	7.79 ± 2.84^**#**^	25.31 ± 9.57*^‡^	37.27 ± 1.83^**#**^	1.46 ± 0.11^**#**^

Values expressed as means (±SD) of 7 (E) - 30 (C) animals per group. All values were computed in ten random, non-coincident fields per mice. Fractional area of normal, collapsed, and hyperinflated alveoli. Lm, mean linear intercept. In the control (C) group, saline was instilled intratracheally. In the emphysema (E) groups, mice received porcine pancreatic elastase intratracheally. At day 21, all groups were randomized to receive saline and bone marrow (BM), adipose (AD), or lung (L)-derived mesenchymal stem cells (MSC, 1×10^5^ cells) intravenously (IV) or intratracheally (IT). *Vs. C group (p < 0.05). ^**#**^Vs. E-SAL group (p < 0.05). ^**†**^Vs. BM-MSC-IV group (p < 0.05). ^**‡**^Vs. BM-MSC-IT group (p < 0.05).

**Table 4:** **Semiquantitative analysis of electron microscopy and apoptosis (TUNEL)**

			**Alveolar-capillary membrane**	**Endothelial cell lesion**	**Pneumocyte II lesion**	**Apoptosis**
C			1 (0–1)	0 (0–1)	1 (0.5–1)	0.5 (0–1)
E	SAL		4 (3–4)*	4 (3–4)*	3 (3–4)*	3.5 (3–4)*
	BM–MSC	IV	2 (1.5–2.5)*^#^	2 (2–2.5)*^#^	2 (1.5–2.5)*^#^	1 (1–1.75)^**#**^
	AD–MSC		2 (1.5–2)*^#^	2 (1.5–2.5)*^#^	2 (2–2.5)*^#^	1.5 (1–2)^**#**^
	BM–MSC	IT	2 (2–2.5)*^#^	2 (2–2.5)*^#^	3 (2–3)*^#^	1 (1–1.75)^**#**^
	AD–MSC		2 (1–2)*^#^	2 (2–3)*^#^	2 (2–2.5)*^#^	1 (−1.75)^**#**^
	L–MSC		2 (2–2.5)*^#^	3 (2.5–3)*^#^	2 (2–3)*^#^	2 (2–2)*^#^

Values expressed as median (interquartile range) of 4 (E) to 16 (C) animals per group. Pathological findings were graded on a five-point, semiquantitative, severity-based scoring system: 0 = normal lung parenchyma, 1 = changes in 1–25%, 2 = 26–50%, 3 = 51–75%, and 4 = 76–100% of the examined tissue in control (C) and emphysema (E) female C57BL/6 mice treated with saline (SAL) or bone marrow (BM), adipose (AD), or lung (L)-derived mesenchymal stem cells (MSC) intravenously (IV) or intratracheally (IT). *Vs. C group (p < 0.05). ^**#**^Vs. E-SAL group (p < 0.05).

**Table 5:** **Lung mechanics**

**Groups**			**Est (cmH**_**2**_ **O.ml** ^**−1**^ **)**
C			30.26 ± 2.27
E	SAL		34.19 ± 6.86
	BM-MSC	IV	34.24 ± 4.07
	AD-MSC		31.37 ± 4.96
	BM-MSC	IT	34.76 ± 1.97
	AD-MSC		35.66 ± 5.39
	L-MSC		29.62 ± 4.00

Static lung elastance (Est, L) at day 28. In the control (C) group, saline was instilled intratracheally once a week for 1 month. Emphysema (E) animals received porcine pancreatic elastase intratracheally following the same protocol. After the last instillation, all groups were randomized to receive saline (SAL) and mesenchymal stem cells (MSC, 1×10^5^ cells) derived from bone marrow (BM), adipose tissue (AD), or lung tissue (LD) intravenously (IV) or intratracheally (IT). Values are means ± SD of 7 (E) – 30 (C) animals in each group (10 determinations per animal).

**Figure 8:**
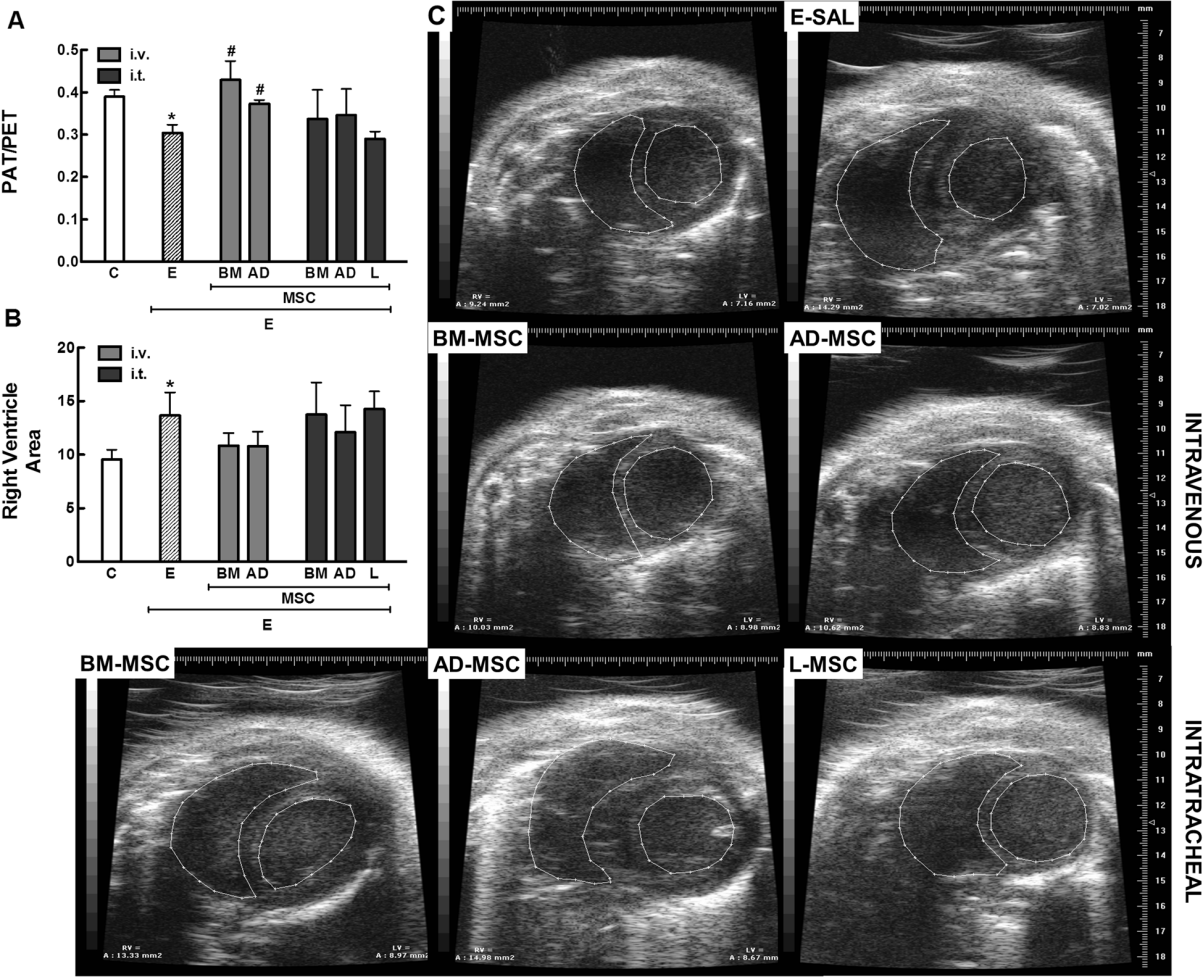
**Short-axis B-dimensional views of both ventricles.** The indices shown are **(** **A** **)** pulmonary artery acceleration time/pulmonary artery ejection time ratio – PAT/PET ratio – and **(** **B** **)** right ventricle area. **(C)** Echocardiographic images. LV: left ventricle, RV: right ventricle. The bars represent the means of 3 mice ± SD/group. C: control groups. E: emphysema groups. Mice were treated with saline (SAL), or bone marrow (BM), adipose (AD) and lung (L)-derived mesenchymal stem cells (MSC). IV: intravenous route. IT: intratracheal route. *Vs. C group (p < 0.05). ^#^Vs. E-SAL group (p < 0.05).

Echocardiography showed increased right ventricle area and reduced pulmonary artery acceleration time–pulmonary artery ejection time (PAT/PET) ratio, an indirect index of pulmonary arterial hypertension (Figure 8), in the E-SAL group compared to controls.

### Delivery of different sources of MSCs

#### Intravenous route

IV delivery of BM- and AD-MSCs led to a similar reduction in Lm, fractional area of alveolar collapse, neutrophil infiltration (Table 3, Figure 2), collagen fiber content around the small airways (Figure 3), and number of apoptotic cells (Table 4), and an increase in the amount of elastic fibers (Figure 4) compared to the E-SAL group. BM- and AD-MSCs attenuated ultrastructural damage of the alveolar-capillary membrane, as well as epithelial and endothelial cells (Table 4, Figure 5). BM- and AD-MSCs reduced the number of macrophages with the M1 immunophenotype (Figure 6A), but only BM-MSCs increased the number of macrophages with the M2 immunophenotype in lung parenchyma (Figure 6B). BM- and AD-MSCs decreased KC and TGF-β levels, but increased VEGF levels in lung tissue (Figure 7). BM- and AD-MSCs did not modify Est, L (Table 5). BM- and AD-MSCs led to a significant reversion of the PAT/PET ratio (Figure 8A) and tended to normalize right ventricle area (Figure 8B).

#### Intratracheal route

IT administration of BM-, AD-, and L-MSCs reduced Lm and neutrophils (Table 3) while increasing elastic fiber content (Figure 4). AD- and L-MSCs reduced the fractional area of alveolar collapse, whereas BM-MSCs decreased the fractional area of lung hyperinflation (Table 3). Although BM-MSCs, AD-MSCs, and L-MSCs reduced collagen fiber deposition around the small airways, BM- and L-MSCs were more effective than AD-MSC (Figure 3). IT administration of all three types of MSCs attenuated ultrastructural damage of the alveolar-capillary membrane, as well as type II epithelial and endothelial cells (Table 4, Figure 5). The number of macrophages with the M2 immunophenotype was higher after administration of BM-MSCs compared to AD-MSCs (Figure 6B). All three cell sources reduced TGF-β levels, but only BM- and L-MSCs significantly decreased KC levels (Figure 7). BM-, AD-, and L-MSCs did not modify Est, L (Table 5). PAT/PET and right ventricle area were not affected by any of the studied MSCs when administered via the IT route (Figure 8).

## Discussion

To our knowledge, this was the first study to compare the potential therapeutic effects of three different sources of MSCs, delivered through two different administration routes, on lung inflammation and remodeling and on cardiovascular function in experimental emphysema induced by repeated doses of elastase. In contrast to the classical single-dose protocols of elastase-induced emphysema [26], which induce only emphysema-like lesions without systemic [27] or cardiovascular impairment, the present model, developed in our laboratory [11], results in lung histological and ultrastructural changes and cardiac impairment that resemble human emphysema. Using this model, all studied MSC groups (with the exception of the IV L-MSC group), regardless of administration route, exhibited decreased Lm, neutrophil infiltration, and cell apoptosis; increased elastic fiber content; reduced alveolar-capillary membrane and type II epithelial and endothelial cell ultrastructural damage; and decreased KC and TGF-β expression in lung tissue. Therefore, MSC administration can modulate the inflammatory and remodeling processes of emphysema; however, specific beneficial effects can differ depending on MSC source and administration route.

While all MSCs share similar general properties, cells from different sources can exhibit significant differences in anti-inflammatory or regenerative potency depending on the particular injury being addressed [28]. Recent studies have compared the characteristics of adult MSCs from different sources [9,10,17,29], and have demonstrated distinct effects in different experimental models, even when cells have similar proliferation and differentiation capacities [30,31]. The relevant mechanisms whereby different MSCs populations have distinct actions in the same disease model remain unclear. In our study, the beneficial effects of MSCs varied according to source.

BM-MSCs are well-characterized and currently the most widely used [16]; however, they require an invasive harvesting process and have limited availability. Like BM-MSCs [4,7,13], AD-MSCs have also demonstrated promising effects on the maintenance of vascular integrity by secreting anti-apoptotic and pro-angiogenic factors [32], and reduce inflammation in experimental emphysema [6,33]. In adults, these cells are easy to obtain in large quantities by liposuction, which makes them good candidates for therapeutic use and facilitates autologous transplantation [34]. More recently, highly proliferative and clonogenic MSC populations have also been isolated from explants [35] and allografts [36] of adult lung tissue. L-MSCs are immunoprivileged, do not express MHC II or the co-stimulatory molecules CD80 or CD86 [37], and can inhibit T cell-based allorecognition [36], facilitating the success of allogeneic transplants. Additionally, L-MSCs express several basement membrane proteins and growth factors which seem to amplify their retention in the injured tissue [12,14], making them promising candidates for cell-based therapy in lung diseases. However, there is limited information regarding the effects of L-MSCs in experimental emphysema [12,14,35]. In contrast to the present study, Hoffman et al. (13) observed no death after IV delivery of L-MSCs in mice. The reasons for this discrepancy are unclear; one potential explanation is the route (jugular versus tail vein) chosen for cell administration. Since no significant differences between saline and lung fibroblasts were observed in our pilot studies, nor in previous reports [38,39], saline was administered as control instead of mouse fibroblasts in the present investigation.

Reports have demonstrated that a direct pathway of delivery – e.g., IT for lung diseases [24] and intra-myocardial for acute ischemia-reperfusion [40] – may result in greater retention of MSCs in the target tissues. However, in an experimental model of ventilator-induced lung injury, MSCs enhanced recovery and repair regardless of administration route (IT vs. IV) [38]. Recent evidence demonstrated that neither IV nor IT administration of BM-MSC is able to revert lung histology in a single dose protocol of elastase-induced emphysema [41], while in the present study, IT administration of BM-MSCs led to a greater reduction in alveolar hyperinflation than IV delivery. This dissociation between the beneficial effects observed in the present study with IT administration versus those obtained with IV administration may be associated with great loss of alveolar membrane surface area in emphysema, resulting in reduced endothelial cell adhesion molecules [42] and, thus, decreased MSC adhesion.

In our emphysema model, the increase in Lm was associated with neutrophil infiltration of lung tissue and M1 macrophage polarization. Conflicting evidence on the effects of smoking in downregulating M1 macrophages has been published [43-46]. Macrophages can be activated by various extracellular signals to polarize toward either the M1 (inflammatory and antimicrobial) or the M2 (wound repair and inflammation resolution) phenotype. The enhanced M1 polarization observed in lung tissue in our experiment is in line with some reports, which evinced an increase in pro-inflammatory macrophages and a reduction of chemokine ligand 18 (CCL18), a chemokine expressed by alternatively activated macrophages, in the bronchoalveolar lavage fluid of smokers compared to nonsmokers [43,44]. In the present study, M1 activation was similarly inhibited by IV delivery of BM-MSC and AD-MSC. However, only BM-MSC therapy stimulated the M2 phenotype, and more effectively when given IV than when administered IT. The differences in M1 and M2 phenotype observed according to the MSC source and route of administration may be explained by the existence of an “environmental-niche memory” in BM-MSCs and an “epithelial” commitment of AD-MSCs, as described in a previous report [29].

In the present study, BM-, AD-, and L-MSCs seemed to differentially modulate production of some chemokines and growth factors associated with the pathophysiology of emphysema. Increasing evidence demonstrates that the pathogenic changes mediated by MSCs are highly sensitive to the microenvironment to which these cells are exposed. For example, MSC-conditioned media may be a rich source of TGF-β secretion and lead to an increase in collagen gene expression [47]. Conversely, in an experimental model of bleomycin-induced fibrosis, BM-MSCs reduced lung tissue TGF-β levels and soluble collagen in lung extracts [48]. In emphysema, increased TGF-β secretion by epithelial cells [49] is associated with progressive small-airway fibrosis. In our study, a similar reduction of TGF-β levels was observed in all MSC-treated groups, regardless of the delivery route; however, it was not accompanied by equal decrease in deposition of collagen fibers in the small airways. Previous reports demonstrated the ability of MSCs to stimulate VEGF production *in vitro* [13,33] and in vivo [11,13] in experimental models of emphysema. We observed that IV administration of BM-MSC and AD-MSC increased VEGF levels in lung tissue, which was not observed with the IT route. Based on previous studies of cardiac revascularization [50,51], we hypothesize that the systemic injection of BM-MSCs and AD-MSCs results in direct contact with the remaining endothelial cells of the pulmonary vasculature, stimulating them to synthesize VEGF. Intense neutrophilia in the sputum of COPD patients correlates positively with disease severity and high production of IL-8 [52]. This chemokine is released by alveolar macrophages when stimulated by pollutant particles, and is responsible for massive neutrophil recruitment to the lungs. We observed that all MSC therapies similarly reduced KC and neutrophilia in lung tissue.

Only IV administration of BM-MSC and AD-MSC reverted the reduction in the PAT/PET ratio, which may be associated with the inhibition of pulmonary microvasculature muscularization and stimulation of VEGF-induced angiogenesis [4]. Nevertheless, these changes did not result in modifications in right ventricular area, probably due to the timing of analysis and the small number of cells that reach the heart.

Despite the major lung morphology changes induced by our model of emphysema, no significant changes in Est, L were observed. This is in agreement with other studies using different experimental models of emphysema, which showed dissociation between the degree of tissue loss and pulmonary dysfunction [53-55].

Several limitations of this study should be considered: (1) the absence of MSC tracking after IT or IV administration, limiting our knowledge regarding the delivery dynamics of each cell lineage; (2) the experimental period of 5 weeks, which may not be enough to understand the late effects of MSC therapy; and (3) only a few specific cytokines and growth factors were evaluated; a wider range of mediators should be analyzed to provide a more complete understanding of the mechanisms associated with each cell type. Additionally, more extensive analysis of the range of soluble mediators released by each MSC type may provide further information on the different effects noted in this model.

In conclusion, all three MSC sources tested (BM-MSC, AD-MSC and L-MSC), regardless of the administration route (with the exception of the IV L-MSC group), attenuated lung damage in this mouse model of elastase-induced experimental emphysema. Nevertheless, MSCs from different sources exhibited distinct effects on the different aspects of lung and cardiovascular injury, through mechanisms that remain unclear. Further research comparing the effects of different MSC sources and routes of administration is required.

## References

[1] Minai OA, Benditt J, Martinez FJ (2008). Natural history of emphysema. Proc Am Thorac Soc.

[2] **From the Global Strategy for the Diagnosis, Management and Prevention of COPD, Global Initiative for Chronic Obstructive Lung Disease (GOLD).** http://www.goldcopd.org/.

[3] Weiss DJ (2014). Concise review: current status of stem cells and regenerative medicine in lung biology and diseases. Stem Cells.

[4] Huh JW, Kim SY, Lee JH, Lee JS, Van Ta Q, Kim M, Oh YM, Lee YS, Lee SD (2011). Bone marrow cells repair cigarette smoke-induced emphysema in rats. Am J Physiol Lung Cell Mol Physiol.

[5] Kim SY, Lee JH, Kim HJ, Park MK, Huh JW, Ro JY, Oh YM, Lee SD, Lee YS (2012). Mesenchymal stem cell-conditioned media recovers lung fibroblasts from cigarette smoke-induced damage. Am J Physiol Lung Cell Mol Physiol.

[6] Schweitzer KS, Johnstone BH, Garrison J, Rush NI, Cooper S, Traktuev DO, Feng D, Adamowicz JJ, Van Demark M, Fisher AJ, Kamocki K, Brown MB, Presson RG, Broxmeyer HE, March KL, Petrache I (2011). Adipose stem cell treatment in mice attenuates lung and systemic injury induced by cigarette smoking. Am J Respir Crit Care Med.

[7] Zhen G, Xue Z, Zhao J, Gu N, Tang Z, Xu Y, Zhang Z (2010). Mesenchymal stem cell transplantation increases expression of vascular endothelial growth factor in papain-induced emphysematous lungs and inhibits apoptosis of lung cells. Cytotherapy.

[8] Weiss DJ, Casaburi R, Flannery R, LeRoux-Williams M, Tashkin DP (2013). A placebo-controlled, randomized trial of mesenchymal stem cells in COPD. Chest.

[9] Ostanin AA, Petrovskii YL, Shevela EY, Chernykh ER (2011). Multiplex analysis of cytokines, chemokines, growth factors, MMP-9 and TIMP-1 produced by human bone marrow, adipose tissue, and placental mesenchymal stromal cells. Bull Exp Biol Med.

[10] Ricciardi M, Malpeli G, Bifari F, Bassi G, Pacelli L, Nwabo Kamdje AH, Chilosi M, Krampera M: **Comparison of epithelial differentiation and immune regulatory properties of mesenchymal stromal cells derived from human lung and bone marrow.** *PLoS One* 2012, **7**(5):e35639. 10.1371/journal.pone.0035639 PMC3342330 22567106

[11] Cruz FF, Antunes MA, Abreu SC, Fujisaki LC, Silva JD, Xisto DG, Maron-Gutierrez T, Ornellas DS, Sa VK, Rocha NN, Capelozzi VL, Morales MM, Rocco PR (2012). Protective effects of bone marrow mononuclear cell therapy on lung and heart in an elastase-induced emphysema model. Respir Physiol Neurobiol.

[12] Hoffman AM, Paxson JA, Mazan MR, Davis AM, Tyagi S, Murthy S, Ingenito EP (2011). Lung-derived mesenchymal stromal cell post-transplantation survival, persistence, paracrine expression, and repair of elastase-injured lung. Stem Cells Dev.

[13] Guan XJ, Song L, Han FF, Cui ZL, Chen X, Guo XJ, Xu WG (2013). Mesenchymal stem cells protect cigarette smoke-damaged lung and pulmonary function partly via VEGF-VEGF receptors. J Cell Biochem.

[14] Ingenito EP, Tsai L, Murthy S, Tyagi S, Mazan M, Hoffman A (2012). Autologous lung-derived mesenchymal stem cell transplantation in experimental emphysema. Cell Transplant.

[15] da Silva Meirelles L, Chagastelles PC, Nardi NB (2006). Mesenchymal stem cells reside in virtually all post-natal organs and tissues. J Cell Sci.

[16] Dominici M, Le Blanc K, Mueller I, Slaper-Cortenbach I, Marini F, Krause D, Deans R, Keating A, Prockop D, Horwitz E (2006). Minimal criteria for defining multipotent mesenchymal stromal cells. The International Society for cellular therapy position statement. Cytotherapy.

[17] Nora CC, Camassola M, Bellagamba B, Ikuta N, Christoff AP, Meirelles Lda S, Ayres R, Margis R, Nardi NB (2012). Molecular analysis of the differentiation potential of murine mesenchymal stem cells from tissues of endodermal or mesodermal origin. Stem Cells Dev.

[18] Lang RM, Bierig M, Devereux RB, Flachskampf FA, Foster E, Pellikka PA, Picard MH, Roman MJ, Seward J, Shanewise J, Solomon S, Spencer KT, St John Sutton M, Stewart W (2006). Recommendations for chamber quantification. Eur J Echocardiogr.

[19] Abbas AE, Franey LM, Marwick T, Maeder MT, Kaye DM, Vlahos AP, Serra W, Al-Azizi K, Schiller NB, Lester SJ (2013). Noninvasive assessment of pulmonary vascular resistance by doppler echocardiography. J Am Soc Echocardiogr.

[20] Thibault HB, Kurtz B, Raher MJ, Shaik RS, Waxman A, Derumeaux G, Halpern EF, Bloch KD, Scherrer-Crosbie M (2010). Noninvasive assessment of murine pulmonary arterial pressure: validation and application to models of pulmonary hypertension. Circ Cardiovasc Imaging.

[21] Hsia CC, Hyde DM, Ochs M, Weibel ER (2010). Structure AEJTFoQAoL: an official research policy statement of the American Thoracic Society/European Respiratory Society: standards for quantitative assessment of lung structure. Am J Respir Crit Care Med.

[22] Weibel ER, Gil J (1990). Morphometry: Stereological Theory and Practical Methods. Models of Lung Disease-Microscopy and Structural Methods.

[23] Antunes MA, Abreu SC, Silva AL, Parra-Cuentas ER, Ab'Saber AM, Capelozzi VL, Ferreira TP, Martins MA, Silva PM, Rocco PR (2010). Sex-specific lung remodeling and inflammation changes in experimental allergic asthma. J Appl Physiol.

[24] Abreu SC, Antunes MA, Maron-Gutierrez T, Cruz FF, Ornellas DS, Silva AL, Diaz BL, Ab'Saber AM, Capelozzi VL, Xisto DG, Morales MM, Rocco PR (2013). Bone marrow mononuclear cell therapy in experimental allergic asthma: intratracheal versus intravenous administration. Respir Physiol Neurobiol.

[25] Oliveira GP, Oliveira MB, Santos RS, Lima LD, Dias CM, Ab’ Saber AM, Teodoro WR, Capelozzi VL, Gomes RN, Bozza PT, Pelosi P, Rocco PR: **Intravenous glutamine decreases lung and distal organ injury in an experimental model of abdominal sepsis.** *Crit Care* 2009, **13**(3):R74. 10.1186/cc7888 PMC2717436 19454012

[26] Antunes MA, Rocco PR (2011). Elastase-induced pulmonary emphysema: insights from experimental models. An Acad Bras Cienc.

[27] Luthje L, Raupach T, Michels H, Unsold B, Hasenfuss G, Kogler H, Andreas S: **Exercise intolerance and systemic manifestations of pulmonary emphysema in a mouse model.** *Respir Res* 2009, **10:**7. 10.1186/1465-9921-10-7 PMC2644670 19175913

[28] Moodley Y, Vaghjiani V, Chan J, Baltic S, Ryan M, Tchongue J, Samuel CS, Murthi P, Parolini O, Manuelpillai U: **Anti-inflammatory effects of adult stem cells in sustained lung injury: a comparative study.** *PLoS One* 2013, **8**(8):e69299. 10.1371/journal.pone.0069299 PMC3731305 23936322

[29] Ragni E, Montemurro T, Montelatici E, Lavazza C, Vigano M, Rebulla P, Giordano R, Lazzari L (2013). Differential microRNA signature of human mesenchymal stem cells from different sources reveals an “environmental-niche memory” for bone marrow stem cells. Exp Cell Res.

[30] Elman JS, Li M, Wang F, Gimble JM, Parekkadan B: **A comparison of adipose and bone marrow-derived mesenchymal stromal cell secreted factors in the treatment of systemic inflammation.** *J Inflamm* 2014, **11**(1):1. 10.1186/1476-9255-11-1 PMC3895743 24397734

[31] Rasmussen JG, Frobert O, Holst-Hansen C, Kastrup J, Baandrup U, Zachar V, Fink T, Simonsen U (2012). Comparison of human adipose-derived stem cells and bone marrow-derived stem cells in a myocardial infarction model. Cell Transplant.

[32] Rajashekhar G, Traktuev DO, Roell WC, Johnstone BH, Merfeld-Clauss S, Van Natta B, Rosen ED, March KL, Clauss M (2008). IFATS collection: adipose stromal cell differentiation is reduced by endothelial cell contact and paracrine communication: role of canonical Wnt signaling. Stem Cells.

[33] Shigemura N, Okumura M, Mizuno S, Imanishi Y, Nakamura T, Sawa Y (2006). Autologous transplantation of adipose tissue-derived stromal cells ameliorates pulmonary emphysema. Am J Transplant.

[34] Antunes MA, Laffey JG, Pelosi P, Rocco PR (2014). Mesenchymal stem cell trials for pulmonary diseases. J Cell Biochem.

[35] Ingenito EP, Sen E, Tsai LW, Murthy S, Hoffman A (2010). Design and testing of biological scaffolds for delivering reparative cells to target sites in the lung. J Tissue Eng Regen Med.

[36] Jarvinen L, Badri L, Wettlaufer S, Ohtsuka T, Standiford TJ, Toews GB, Pinsky DJ, Peters-Golden M, Lama VN (2008). Lung resident mesenchymal stem cells isolated from human lung allografts inhibit T cell proliferation via a soluble mediator. J Immunol.

[37] Lama VN, Smith L, Badri L, Flint A, Andrei AC, Murray S, Wang Z, Liao H, Toews GB, Krebsbach PH, Peters-Golden M, Pinsky DJ, Martinez FJ, Thannickal VJ (2007). Evidence for tissue-resident mesenchymal stem cells in human adult lung from studies of transplanted allografts. J Clin Invest.

[38] Curley GF, Ansari B, Hayes M, Devaney J, Masterson C, Ryan A, Barry F, O'Brien T, Toole DO, Laffey JG (2013). Effects of intratracheal mesenchymal stromal cell therapy during recovery and resolution after ventilator-induced lung injury. Anesthesiology.

[39] Gupta N, Krasnodembskaya A, Kapetanaki M, Mouded M, Tan X, Serikov V, Matthay MA (2012). Mesenchymal stem cells enhance survival and bacterial clearance in murine Escherichia coli pneumonia. Thorax.

[40] Bonios M, Terrovitis J, Chang CY, Engles JM, Higuchi T, Lautamaki R, Yu J, Fox J, Pomper M, Wahl RL, Tsui BM, O’Rourke B, Bengel FM, Marbán E, Abraham MR (2011). Myocardial substrate and route of administration determine acute cardiac retention and lung bio-distribution of cardiosphere-derived cells. J Nucl Cardiol.

[41] Tibboel J, Keijzer R, Reiss I, de Jongste JC, Post M (2014). Intravenous and intratracheal mesenchymal stromal cell injection in a mouse model of pulmonary emphysema. COPD.

[42] Nystedt J, Anderson H, Tikkanen J, Pietila M, Hirvonen T, Takalo R, Heiskanen A, Satomaa T, Natunen S, Lehtonen S, Hakkarainen T, Korhonen M, Laitinen S, Valmu L, Lehenkari P (2013). Cell surface structures influence lung clearance rate of systemically infused mesenchymal stromal cells. Stem Cells.

[43] Frankenberger M, Menzel M, Betz R, Kassner G, Weber N, Kohlhaufl M, Haussinger K, Ziegler-Heitbrock L (2004). Characterization of a population of small macrophages in induced sputum of patients with chronic obstructive pulmonary disease and healthy volunteers. Clin Exp Immunol.

[44] Kollert F, Probst C, Muller-Quernheim J, Zissel G, Prasse A (2009). CCL18 production is decreased in alveolar macrophages from cigarette smokers. Inflammation.

[45] Kunz LI, Lapperre TS, Snoeck-Stroband JB, Budulac SE, Timens W, van Wijngaarden S, Schrumpf JA, Rabe KF, Postma DS, Sterk PJ, Hiemstra PS, Groningen Leiden Universities Corticosteroids in Obstructive Lung Disease Study Group: **Smoking status and anti-inflammatory macrophages in bronchoalveolar lavage and induced sputum in COPD.** *Respir Res* 2011, **12:**34. 10.1186/1465-9921-12-34 PMC3072953 21426578

[46] Shaykhiev R, Krause A, Salit J, Strulovici-Barel Y, Harvey BG, O'Connor TP, Crystal RG (2009). Smoking-dependent reprogramming of alveolar macrophage polarization: implication for pathogenesis of chronic obstructive pulmonary disease. J Immunol.

[47] Salazar KD, Lankford SM, Brody AR (2009). Mesenchymal stem cells produce Wnt isoforms and TGF-beta1 that mediate proliferation and procollagen expression by lung fibroblasts. Am J Physiol Lung Cell Mol Physiol.

[48] Lee SH, Jang AS, Kim YE, Cha JY, Kim TH, Jung S, Park SK, Lee YK, Won JH, Kim YH, Park CS: **Modulation of cytokine and nitric oxide by mesenchymal stem cell transfer in lung injury/fibrosis.** *Respir Res* 2010, **11:**16. 10.1186/1465-9921-11-16 PMC2827393 20137099

[49] Takizawa H, Tanaka M, Takami K, Ohtoshi T, Ito K, Satoh M, Okada Y, Yamasawa F, Nakahara K, Umeda A (2001). Increased expression of transforming growth factor-beta1 in small airway epithelium from tobacco smokers and patients with chronic obstructive pulmonary disease (COPD). Am J Respir Crit Care Med.

[50] Rodrigues CG, Plentz RD, Dipp T, Salles FB, Giusti II, Sant’anna RT, Eibel B, Nesralla IA, Markoski M, Beyer NN, Kalil RA (2013). VEGF 165 gene therapy for patients with refractory angina: mobilization of endothelial progenitor cells. Arq Bras Cardiol.

[51] Ye J, Ni P, Kang L, Xu B (2012). Apelin and vascular endothelial growth factor are associated with mobilization of endothelial progenitor cells after acute myocardial infarction. J Biomed Res.

[52] Keatings VM, Collins PD, Scott DM, Barnes PJ (1996). Differences in interleukin-8 and tumor necrosis factor-alpha in induced sputum from patients with chronic obstructive pulmonary disease or asthma. Am J Respir Crit Care Med.

[53] Bates JH, Davis GS, Majumdar A, Butnor KJ, Suki B (2007). Linking parenchymal disease progression to changes in lung mechanical function by percolation. Am J Respir Crit Care Med.

[54] Hamakawa H, Bartolak-Suki E, Parameswaran H, Majumdar A, Lutchen KR, Suki B (2011). Structure-function relations in an elastase-induced mouse model of emphysema. Am J Respir Cell Mol Biol.

[55] Winkler T, Suki B (2011). Emergent structure-function relations in emphysema and asthma. Crit Rev Biomed Eng.

